# Determination of absorbed dose to water for high-energy photon and electron beams-comparison of the standards DIN 6800-2 (1997), IAEA TRS 398 (2000) and DIN 6800-2 (2006)

**DOI:** 10.4103/0971-6203.31143

**Published:** 2007

**Authors:** Golam Abu Zakaria, Wilhelm Schuette

**Affiliations:** Department of Medical Radiation Physics, Kreiskrankenhaus Gummersbach, Academic Teaching Hospital of the University of Cologne, Wilhelm-Breckow-Allee 20, 51643 Gummersbach, Germany

**Keywords:** Absorbed dose to water, DIN 6800-2 protocol (1997), DIN 6800-2 protocol (Draft National Standard 2006), external beam photons and electrons, IAEA TRS-398 protocol (2000)

## Abstract

For the determination of the absorbed dose to water for high-energy photon and electron beams the IAEA code of practice TRS-398 (2000) is applied internationally. In Germany, the German dosimetry protocol DIN 6800-2 (1997) is used. Recently, the DIN standard has been revised and published as Draft National Standard DIN 6800-2 (2006). It has adopted widely the methodology and dosimetric data of the code of practice. This paper compares these three dosimetry protocols systematically and identifies similarities as well as differences. The investigation was done with 6 and 18 MV photon as well as 5 to 21 MeV electron beams. While only cylindrical chambers were used for photon beams, measurements of electron beams were performed using cylindrical as well as plane-parallel chambers. The discrepancies in the determination of absorbed dose to water between the three protocols were 0.4% for photon beams and 1.5% for electron beams. Comparative measurements showed a deviation of less than 0.5% between our measurements following protocol DIN 6800-2 (2006) and TLD inter-comparison procedure in an external audit.

The determination of the absorbed dose to water for high-energy photon and electron beams is performed in Germany according to the German dosimetry protocol DIN 6800-2 (1997).[1] This protocol is based on the use of ionization chambers calibrated in terms of absorbed dose to water in a cobalt-60 gamma radiation beam. The protocol has been revised by a German task group and published as “Draft National Standard (DNS)” DIN 6800-2 (2006) in March 2006.[2] The new version has adopted widely the methodology and dosimetric data of the Code of practice TRS-398 published in 2000.[3] The main part of the revised protocol consists of the measurement procedures, parameters for various chambers and the evaluation of uncertainties. The physical backgrounds are described in detail in the appendix of the protocol.

The DIN 6800-2 (DNS 2006) took over the data of IAEA TRS 398 for the calculation of correction factors and interaction coefficients and therefore has improved its consistency with the international practice of dose determinations. But the DIN 6800-2 (DNS 2006) did not simply take over the said data; it considered the clinical demands of Germany. The gist of its changes lies in the electron dosimetry and in the introduction of the evaluation of uncertainties in measurements.

In an earlier work we compared the DIN 6800-2 (1997) to the protocols AAPM TG 51[4] and IAEA TRS 398 and presented the results in detail.[5] In this work the clinical application of DIN 6800-2 (DNS 2006) has been investigated in comparison to the previous protocol DIN 6800-2 (1997) as well as to protocol IAEA TRS 398 as the basis of the future improvements.

## Materials and Methods

The measurements were performed in a Wellhoefer water phantom (*blue* phantom). Depth dose distributions (depth ionization curves) were measured with a Wellhoefer CC-13 and a plane-parallel type (PTW-34001 *Roos* chamber) ionization chamber. We used the plane-parallel *Roos* chamber to make accurate measurements in the build-up region. Absolute dosimetry in terms of absorbed dose to water has been performed with a cylindrical (PTW-M31013 chamber) and a plane-parallel PTW-34001 (*Roos* chamber) ionization chamber. All ionization chambers used and their characteristics are listed in Table 1.

**Table 1:** Characteristics of the ionization chambers

*Chamber*	*PTW-31013 (Cylinder)*	*PTW-30006 (Farmer)*	*PTW-23343 (Markus)*	*PTW-34001 (Roos)*
Outer electr.-Ø	5.5 mm	6.1 mm	-	-
Inner electr.-Ø	1.0 mm	1.1 mm	-	-
Wall material	PMMA	PMMA + graphit	-	-
Wall thickness	0.75 mm	0.335 mm	-	-
Electrode spacing	-	-	2.0 mm	2.0 mm
Chamber-Ø	-	-	6.0 mm	15.0 mm
Membrane material	-	-	Polyaethylen	PMMA
Membrane thickness	-	-	0.03 mm	1.0 mm
Guard ring width	-	-	4 mm	0.2 mm
Cavity volume	0.3 cm^3^	0.6 cm^3^	0.055 cm^3^	0.35 cm^3^
Voltage	400V	400V	300V	100V

The relative measurements were done and evaluated by Scanditronix-Wellhoefer software *OmniPro-Accept 6.3*. A Scanditronix-Wellhoefer chamber served as reference chamber. The absolute charge was measured with the electrometer models UNIDOS and UNIDOS E (PTW Freiburg). The operating voltage for the cylindrical chamber PTW-31013 was 400V, whereas for the Roos chamber the voltage was 100V.

All the measurements were performed under reference conditions. The reference point of the cylindrical chamber in the phantom was positioned according to the reference condition of each protocol. For plane-parallel chamber the reference point is taken to be on the inner surface of the entrance window, at the centre of the window. Reference conditions for the determination of absorbed dose to water in high-energy photon and electron beams are given in Table 2.

**Table 2:** Reference conditions for the determination of depth dose curve and absorbed dose to water in high-energy photon and electron beams (z_ref_ = reference depth) for all three protocols. IAEA recommends a field size of 10×10 cm^2^ for electron beams, but we have chosen a field size of 20×20 cm^2^ for all three protocols for comparison

	*Depth dose distribution*		*Absorbed dose to water*	
		
	*Photons*	*Electrons*	*Photons*	*Electrons*
Field size at				
SSD:	10×10 cm^2^	20×20 cm^2^	10×10 cm^2^	20×20 cm^2^
SSD:	100 cm	100 cm	100 cm	100 cm
Measurement depth:	-	-	z_ref_ = 10 cm	z_ref_ = 0.6*R_50_ - 0.1

The irradiation units used are the Siemens linear accelerators, *ONCOR Impression* (6 MV photon and 5, 7, 8, 10, 12, 14 MeV electron beams, assigned as Acc. 1) and *ONCOR Avant garde* (6 and 18 MV photon and 6, 9, 12, 15, 18 and 21 MeV electron beams, assigned as Acc. 2).

Table 3 represents the possible ranges of application for different beam qualities and chambers in each of the dosimetry protocols.

**Table 3:** Possible ranges of application for different beam qualities and chambers in each dosimetry protocol

*Dosimetry protocol Beam quality*	*DIN 6800-2 (1997)*		*IAEA 398 (2000)*		*DIN 6800-2 (DNS 2006)*	
		
	*Photons*	*Electrons*	*Photons*	*Electrons*	*Photons*	*Electrons*
Cylindrical chamber PTW-31013 (*Flexible* chamber)	X	recommended ≥ 10 MeV	X	≥ 10 MeV	X	recommended ≥10 MeV
Cylindrical chamber PTW-30006 (*Farmer* chamber)	X	recommended ≥ 10 MeV	X	≥ 10 MeV	X	recommended ≥ 10 MeV
Plane-parallel PTW -34001 (*Roos* chamber)	not re-commended	X	not re-commended	X	not re-commended	X
Plane-parallel PTW-23343 (*Markus* chamber)	not permitted	not permitted	not re-commended	X	not re-commended	not re-commended

X = (here) unrestricted application

The absorbed dose to water for high-energy photon or electron beams is calculated by the following general formula (1):

(1) $$D_{W}\left(z\right)\;=\;M\left(z\right)\;N_{W}\;k_{P}\;k_{S}\;k_{\rho }\;k_{Q}^{{}^{\circ}}\left(z\right)\;$$

where D_w_ = absorbed dose to water

z = depth of measurement

M = reading of the dosimeter

N_w_ = calibration factor for absorbed dose to water for cobalt 60 beams

k_p_ = polarity correction factor

k_s_ = ion recombination factor

k_ρ_ = air density correction

k_Q_° = an “equivalent” quality correction factor in all three protocols

The procedure of the determination of the correction factors k_p_ and k_s_ as well as the quality correction factor k_Q_° are different in each of these protocols. The quality correction factor is designated in the general formula for all the three protocols by an equivalent term k_Q_°. Other factors (such as the gradient correction factor, the perturbation correction factor, etc.) are considered in the formula of each protocol separately. Although the above formalism allows the determination of absorbed dose for electron beams by the use of plane-parallel chambers calibrated in cobalt beams, it is recommended that the plane-parallel chambers are calibrated in a high-energy electron beam against a reference cylindrical chamber calibrated in a Co-60 beam (cross-calibration). This is because we don't know the exact values for perturbation correction factors (wall correction factors) at a cobalt beam for plane-parallel chambers.

Table 4 briefly shows the reference conditions and the formalism for the determination of the quality correction factors in the respective protocol where the different terms used are described in the legend of the table. Further details of practical procedures in the determination of correction factors are described below.

**Table 4:** The reference conditions and the formalism of determination of the quality correction factors for photon beams k_Q_ and electron beams k_E_(z_ref_) in different protocols (k_r_ = gradient correction factor, eff. point of measurement: the effective point of measurement is located at reference depth z_ref_, ref. point of chamber axis: the effective point of measurement is located at z_ref_ + 0.5*r_cav_ [r_cav_ = inner radius of the sensitive volume])

*Dosimetry protocol Radiation Reference depth z_ref_*	*DIN 6800-2 (1997)*		*IAEA 398 (2000)*		*DIN 6800-2 (DNS 2006)*	
		
	*Photons*	*Electrons*	*Photons*	*Electrons*	*Photons*	*Electrons*
	*10 cm*	0.6*R_50_ - 0.1 cm	*10 cm*	0.6*R_50_ - 0.1 cm	*10 cm*	0.6*R_50_ - 0.1 cm
Cylindrical chamber	k_Q_° = k_Q_*k_r_	k_Q_° = k_E_'*k_E_”*k_r_	k_Q_° = k_Q_	k_Q_° = k_Q_	k_Q_° = k_Q_*k_r_	k_Q_° = k_E_' k_E_” *k_r_
	k_Q_ from table 4	k_E_' = f (R_50_,R_p_) k_E_” = p_E_/p_Co_	k_Q_ from Table 14	k_Q_ from Table 18	k_Q_ from Table 6	k_E_' = f (R_50_) k_E_” from Table 8
	eff. point of measurement	eff. point of measurement	ref. point of chamber axis	eff. point of measurement	eff. point of measurement	eff. point of measurement
Plane-parallel chamber	-	k_Q_° = k_E_'*k_E_”	-	k_Q_° = K_Q_	-	k_Q_° = k_E_'*k_E_”
	-	k_E_' = f (R_50_,R_p_)	-	k_Q_ from Table 19	-	k_E_' = f (R_50_)
	-	k_E_” from cross calibration ref. point of chamber axis	-	N_w_ from cross calibration ref. point of chamber axis	-	k_E_” from Table 9 ref. point of chamber axis

For the purpose of this work we have participated in an external audit (Messtechnische Kontrolle, MTK) based on measurements with thermoluminescent dosemeters (TLD).[6] The dosimetric intercomparison measurements were done in a special type of water phantom PTW-4322 with TLD in combination with a cylindrical chamber for photon beams and with a *Roos* chamber for electron beams.

## Determination of the individual correction factors

### Air density correction factor

Air density correction factor k_ρ_ can be determined directly from the temperature and pressure at the measuring spot (t and p method) or by using the check source method (the reference values of the temperature = 293.3 K and pressure = 1013.25 hPa). The check source is heavily shielded. Thus, it takes a long time to achieve the surrounding temperature. Moreover, the temperature of the water phantom is not necessarily the same as the temperature of the surrounding air. Our experience showed that a deviation of up to 1% occurred between the two methods. Therefore we preferred to use the t and p method for our measurements. The air density correction factor is given by the equation (2):

(2) kρ = 273.2+t/c0293.2*1013.25p/hPa

where t = temperature at measuring phantom and p = current air pressure at measuring spot.

### Ion recombination correction factor k_s_

The incomplete collection of charge due to ion recombination in an ionization chamber requires correction factor k_s_. This factor can be measured or calculated by different empirical formula depending on radiation and chamber types. The correction factor is determined by using the following methods given in the protocols for pulsed beams.

DIN 6800-2 (1997)

Theoretical

(3) $$k_{s}=\;1\;+\;0.54\;*\;\mathrm{Di}\;*\;d^{2}/U_{1}$$

IAEA TRS 398 (2000)

Experimental

(4) $$k_{s}=\frac{M_{1}/M_{2}\;-\;1}{U_{1}/U_{2}\;-\;1}$$

DIN 6800-2 (DNS 2006)

Theoretical

(5) $$k_{s}=1\;+\;\left(\gamma +\delta \;*\;D_{i}\right)/U_{1}$$

where:

d = electrode distance

D_i_ = dose per radiation pulse in mGy

U_1_ = normal chamber operating voltage

U_2_ = lower chamber voltage

M_1_ = measured values at U_1_

M_2_ = measured values at U_2_

γ and δ constants taken from DIN 6800-2 (DNS 2006) in Table 4.

For the determination of k_s_ for the DIN 6800-2 (1997) protocol, the *Derikum* formula

(6) $$k_{s}=1\;+\;\left(0.12\;+\;0.46*D_{i}*d^{2}\right)$$

has been used,[7] because the *Boag* formula given in the DIN 6800-2 (1997) does not account for chambers with shorter electrode distance like the *Roos* chamber. The maximal deviation between the values calculated by DIN 6800-2 (DNS 2006) and *Derikum* is approximately 0.5%. The dose per radiation pulse D_i_ can be calculated from the pulse-repetition-frequency PRF and the dose rate DL during a pulse in Gy/min under reference condition:

(7) $$D_{i}\;=\;\left(\mathrm{DL}\;*\;\mathrm{PRF}\right)/60$$

### The polarity effect correction factor

For the determination of k_p_ according to the recommendation of the protocols, a ^60^Co gamma radiation source is needed. Many hospitals, like ours for example, do not have any ^60^Co gamma radiation unit. For the determination of k_P_ we used 6MV Photon beams instead of ^60^Co gamma radiation using the following formulas:

DIN 6800-2 (1997)

(8) $$k_{p}\;=\;\left(M_{1}\;+\;M_{2}\right)/M_{1}/{\left[\left(M_{1}\;+\;M_{2}\right)/M_{1}\right]}_{\mathrm{Co}}$$

IAEA TRS 398 (2000)

(9) $$k_{p}=\left[\frac{M_{+}\;+\;M_{-}}{2M}\right]/{\left[\frac{M_{+}\;+\;M_{-}}{2M}\right]}_{\mathrm{Co}}$$

DIN 6800-2 (DNS 2006)

(10) $$k_{p}\;=\;\left(M_{1}\;+\;M_{2}\right)/M_{1}/{\left[\left(M_{1}\;+\;M_{2}\right)/M_{1}\right]}_{\mathrm{Co}}$$

where M_1_ or M_+_ is the absolute value of reading obtained with the usual polarity and M_2_ or M_-_ the absolute value of reading obtained with the opposite polarity.

### Displacement correction factor k_r_

This correction factor k_r_ is a special case for both German protocols for photon and electron beams. The measurement is always done at an effective point of measurement in the phantom, but calibration refers to the chamber centre. According to both DIN protocols, the effective point of measurement is shifted for cylindrical chambers in photon and electron beams from the chamber axis towards the radiation source, whereas TRS recommends the shift only for electron beams. The correction factor takes into account the different placement effects during calibration and measurement in a beam of high-energy photon and electron radiation. In contrast to TRS-398 the displacement correction factor is not included in the quality correction factor in DIN 6800-2 (1997) as well as DIN 6800-2 (DNS 2006). We have calculated the k_r_ according to DIN 6800-2 (DNS 2006):

(11) $${\text{k}}_{\text{r}}=1+\text{r}/2*\delta$$

where r is the inner radius of the sensitive volume of a cylindrical chamber and δ the relative gradient of the depth dose curve in reference depth during calibration with ^60^Co radiation (for ^60^Co radiation: δ = 0.006 1/mm). The displacement correction factor for the cylindrical chamber PTW-31013 (r = 2.8 mm) is given in Table 5.

**Table 5:** Displacement from the chamber axis towards the radiation source (effective point of measurement) and displacement correction factor for the cylindrical chamber PTW 31013

*Radiation*	*Parameter*	*DIN 6800-2 (1997)*	*DIN 6800-2 (DNS 2006)*	*TRS 398 (2000)*
Photons	Displacement correction factor	k_r_ = 1.007	k_r_ = 1.0084	k_r_ not used
	
	Displacement from the			

	chamber axis	1.4 mm	1.4 mm	0
Electrons	Displacement correction factor	k_r_ = 1.007	k_r_ = 1.0084	k_r_ in k_o_
	
	Displacement from the chamber axis	1.4 mm	1.4 mm	1.4 mm

### Determination of the quality correction factor k_Q_ for the beam quality index Q for photons

The beam quality index Q must be known to determine the quality correction factor k_Q_. The quality index Q for photon beams can be obtained for both protocols DIN 6800-2 (DNS 2006) and IAEA TRS- 398 (2000) from the equation (12):

(12) $$\text{Q}=1.2661*\frac{\text{PDD (d}=20\text{ cm)}}{\text{PDD (d}=10\text{ cm)}}-0.0595$$

where PDD is the percentage depth dose at depth d for a field size of 10 cm × 10 cm defined at the phantom surface with an SSD of 100 cm. Quality index Q is specified by tissue phantom ratio TPR_20,10_ in IAEA TRS- 398.

According to protocol DIN 6800-2 (1997), quality index Q' should first be calculated from the depth dose curve by using the following formula (13):

(13) $${\text{Q}}^{’}=\frac{\text{PDD (d}=10\text{ cm, SSD}=100\text{ cm)}}{\text{PDD (d}=20\text{ cm, SSD}=100\text{ cm)}}$$

The relation between Q and Q' is given by the equation (14) in DIN 6800-2 (1997):

(14) $$Q\;=\;2.012-1.050*Q’+0.1265*Q{’}^{2}+0.01887*Q{’}^{3}$$

Calculated values of the factors k_Q_ are given in Table 6 of DIN 6800-2 (DNS 2006), in Table 14 of IAEA TRS 398 and in Table 4 of DIN 6800-2 (1997) for various cylindrical ionization chambers as a function of beam quality index Q.

**Table 6:** Beam quality Q and beam quality correction factor k_Q_ for different photon energies and protocols

	*DIN 6800-2 (1997)*		*DIN 6800-2 (DNS 2006)*		*TRS398 (2000)*	
	
*Photon energy*	*Q*	*k_Q_*	*Q*	*k_Q_*	*Q*	*k_Q_*
6MV-Photons						
Acc. 1	0.6747	0.9881	0.6751	0.9890	0.6751	0.9912
6MV-Photons						
Acc. 2	0.6701	0.9887	0.6707	0.9896	0.6707	0.9918
18MV-Photons						
Acc. 2	0.7743	0.9655	0.7732	0.9666	0.7732	0.9706

The Data of Table 6 in DIN 6800-2 (DNS 2006) for our cylindrical chamber PTW-31013 were fitted with the following polynomial equation (15) so that we can easily calculate k_Q_ for any Q.

(15) $$k_{Q,31013}=\;0.584322\;+\;3.295307*Q\;-\;9.246571\;*\;Q^{2}\;+\;11.275614*Q^{3}\;-\;5.175615*Q^{4}$$

Similar approximation equations were also fitted for chamber PTW-31013 for TRS 398 and DIN 6800-2 (1997) protocols.

The quality correction factors k_Q_ as a function of beam quality Q for those three protocols are given in Table 6. The values of k_Q_ showed a maximum discrepancy of 0.2% between the protocols. The discrepancy can be explained by the fact that the method of beam quality specification as well as the data used for calculating k_Q_ slightly differ between the protocols.

### Determination of correction factor k_E_ for beam quality index of electrons

According to DIN 6800-2 (1997) the beam quality index for electrons is a function of half-value depth R_50_ and practical range R_p_ in water, whereas only R_50_ characterizes the beam quality index for DIN 6800-2 (DNS 2006) and IAEA TRS 398.

For both German protocols correction factor k_E_ is the product of two factors. The first factor k_E_' is a quality-specific one, it is defined as the quotient of water/air stopping-power ratios at the beam qualities Q and ^60^Co. The second one, k_E_”, is a chamber-specific factor consisting of overall perturbation correction factors for the beam qualities Q and ^60^Co.

On the other hand k_E_ is not split up in protocol TRS- 398. While in DIN 6800-2 (1997) the determination of k_E_' is followed by the virtual energy method developed by Harder.[8] For the determination of k_E_', DIN 6800-2 (DNS 2006) has adopted a method given in TRS 398 (TRS 398, Appendix II).

DIN 6800-2 (1997) recommends to measure the absorbed dose to water for electron beams at the depth of maximum dose or at an energy-dependent reference depth. The users are advised to calibrate a plane-parallel chamber for the use of electron beams against a reference cylinder chamber calibrated in ^60^Co gamma radiation in a higher energy electron beam (cross-calibration). Our reference radiation for cross-calibration was an 21MeV electron beam.[1,5]

In contrast to DIN 6800-2 (1997), DIN 6800-2 (DNS 2006) and the IAEA TRS 398 serve R_50_ as the beam quality index. The half-value of the depth dose distribution in water R_50_ is obtained from the depth ionization distribution, R_50,ion_ using equation (16) (valid R_50,ion_ < 10 cm and corresponds to an energy < 25MeV):

(16) $${\text{R}}_{50}=1.029{\text{ * R}}_{50,\text{ion}}-0.06$$

The reference depth can be obtained from R_50_ given by equation (17):

(17) $${\text{z}}_{\text{ref}}=0.6\;*{\text{ R}}_{50}-0.1$$

Quality-specific factor k_E_' at reference depth z_ref_ can also be calculated following DIN 6800-2 (DNS 2006) using R_50_:

(18) $$k_{E}’\left(z_{\mathrm{ref}}\right)\;=\;1.106\;-\;0.1312\;*\;{R_{50}}^{0.214}$$

For various cylindrical chambers, the chamber-specific factor k_E_” are given in DIN 6800-2 (DNS 2006), Table 7, as a function of beam quality R_50_. The data of our cylindrical chamber PTW-31013 are fitted into the following polynomial so that quality correction factor k_E_” for R_50_ can easily be calculated:

(19) $$k_{E}”\left(z_{\mathrm{ref}}\right){}_{31013}\;=\;0.945103\;+\;0.005644*R_{50}\;-\;0.000202*{R_{50}}^{2}\;+\;0.000002*{R_{50}}^{3}$$

**Table 7:** Influence quantities and their contributions to total uncertainties

*Influence quantities*	*Source*	*Cylindrical chamber Photon beams*	*Cylindrical chamber Electron beams*	*Roos chamber Electron beams*
N_w_	DIN 6800-2 (2006)	0.45	0.45	0.45
Depth of measurement	Estimation	0.1	0.1	0.1
FOA	Estimation	0.1	0.1	0.1
Leakage current	Manufacturer's figure	0.2	0.2	0.2
k_P_	DIN 6800-2 (2006)	0.1	0.1	0.1
k_s_	DIN 6800-2 (2006)	0.1	0.1	0.1
k_ρ_	DIN 6800-2 (2006)	0.17	0.17	0.17
k_Q_ or k_E_	IAEA TRS 398	1.0	0.9	0.6
k_Q_ or k_E_	DIN 6800-2 (2006)	1.0	1.2	1.3
Dosimeter reading	Manual PTW-UNIDOS	0.5	0.5	0.5
Long-term stability Dosimeter/year	Manual PTW-UNIDOS	0.1	0.1	0.1
Total uncertainty according to TRS 398		1.25	1.17	0.96
Total uncertainty according to DIN 6800-2 (2006)		1.25	1.42	1.50

k_E_” for a plane-parallel chamber can be found out from the perturbation correction factor for ^60^Co gamma radiation. For the *Roos* chamber, the value of this factor is according to DIN 6800-2 (DNS 2006), Table 9:

(20) $$k_{E}”\left(z_{\mathrm{ref}}\right){}_{\mathrm{Roos}}\;=\;0.9806$$

According to DIN 6800-2 (DNS 2006) we do not need to perform the time-consuming cross-calibration for most of the plane-parallel chambers. The cross-calibration has to be done for new chambers only.

In TRS 398 protocol, the calculated values of the product of k_E_' * k_E_” (= k_E_, in TRS denoted by k_Q_) are given in Table 18 for a number of chamber types and for a series of beam quality R_50_ at the reference depth. The value for our cylindrical chamber PTW-31013 may be obtained by interpolation or by fitting a similar polynomial as described above.

Like DIN 6800-2 (1997), IAEA recommends cross-calibration for plane-parallel chambers.

For our purpose the highest energy 21MeV was used for cross-calibration to determine the chamber calibration factor for the *Roos* chamber.

The calibration factor N_w_^pp^ can be obtained using equation (21):

(21) $${\mathrm{NN}}_{w,\mathrm{Qcross}}^{\mathrm{pp}}\;=\;\frac{D_{w}^{\mathrm{cc}}}{{\left(k_{p}k_{s}k_{\rho }M\right)}^{\mathrm{pp}}}$$

The absorbed dose to water D_w_ at z_ref_ for cross-calibrated plane-parallel chamber is given by:

(22) $${\text{D}}_{\text{w}}^{\text{pp}}={\text{k}}_{\text{p}}{\text{k}}_{\text{s}}{\text{k}}_{\text{ρ}}\frac{{\text{k}}_{\text{Q,Qint}}}{{\text{k}}_{\text{Qcross,Qint}}}{\text{N}}_{\text{w,Qcross}}^{\text{pp}}\text{M}$$

with the values of k_Q_ for the calculating electron energy and k_Q,cross_ for 21 MeV. These values are given in Table 19 of TRS 398.

We have presented the beam quality correction factors k_E_ for different electron energies following different protocols in Table 8.

**Table 8:** Beam quality R_50_ and beam quality correction factor k_E_ for different electron energies and protocols

*Electron energy*	*R_50_*	*DIN 6800-2 (1997)*		*DIN 6800-2 (DNS 2006)*		*TRS 398 (2000)*	
				
		*Chamber 31013*	*Roos chamber*	*Chamber 31013*	*Roos chamber*	*Chamber 31013*	*Roos chamber*
5MeV Acc. 1	1.85	0.9154	0.9348	0.9131	0.9375	-	1.0611
7MeV Acc. 1	2.58	0.9106	0.9266	0.9059	0.9267	-	1.0502
8MeV Acc. 1	3.04	0.9080	0.9219	0.9024	0.9212	-	1.0440
10MeV Acc. 1	3.92	0.9037	0.9140	0.8970	0.9120	0.9125	1.0335
12MeV Acc. 1	4.66	0.9005	0.9080	0.8933	0.9055	0.9095	1.0259
14MeV Acc. 1	5.26	0.8979	0.9032	0.8908	0.9008	0.9074	1.0205
6MeV Acc. 2	2.32	0.9123	0.9350	0.9083	0.9303	-	1.0539
9MeV Acc. 2	3.46	0.9059	0.9235	0.8996	0.9166	-	1.0388
12MeV Acc. 2	4.59	0.9007	0.9138	0.8937	0.9061	0.9098	1.0266
15 MeV Acc. 2	5.96	0.8943	0.9025	0.8882	0.8958	0.9051	1.0149
18 MeV Acc. 2	7.44	0.8894	0.8929	0.8834	0.8867	0.9012	1.0052
21 MeV Acc. 2	8.45	0.8866	0.8874	0.8805	0.8812	0.8992	1.0000

## Results and Discussion

**Figure 1:**
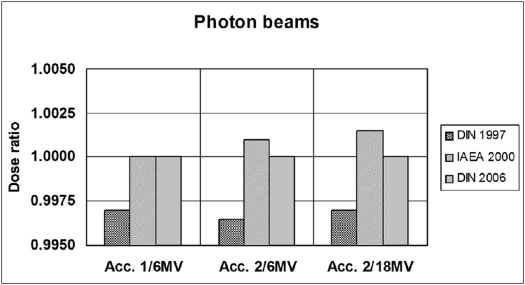
Comparison of absorbed dose ratios for the three protocols in reference to DIN 6800-2 (2006) in photon beams from the linear accelerators *ONCOR Impression* (Acc. 1) and *ONCOR Avant garde* (Acc. 2)

**Figure 2:**
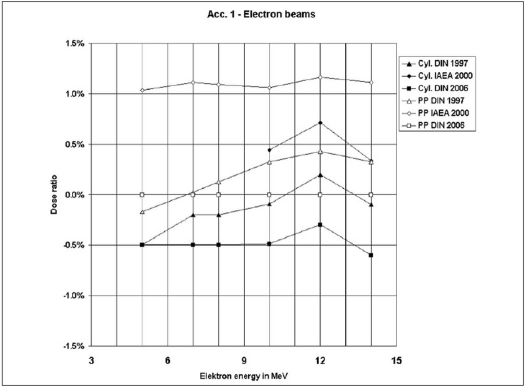
Comparison of absorbed dose ratios for the three protocols in reference to DIN 6800-2 (2006) as a function of electron energy (Linear accelerator Acc. 1)

**Figure 3:**
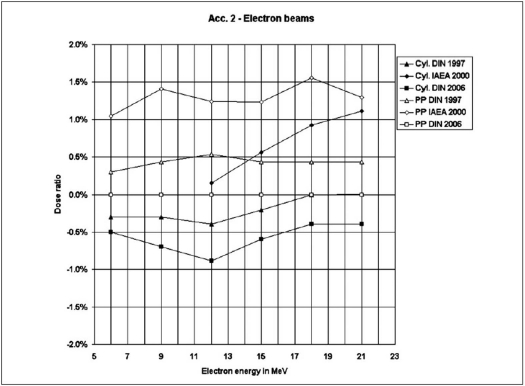
Comparison of absorbed dose ratios for the three protocols in reference to DIN 6800-2 (2006) as a function of electron energy (Linear accelerator Acc. 2)

Figures 1–3 represent the ratios of absorbed doses as a function of beam energies determined by these three dosimetry protocols under reference conditions for photon and electron beams using both chambers. The ratios are obtained by dividing the doses of the protocols by the doses of dosimetry protocol DIN 6800-2 (DNS 2006) for respective energies.

While only cylindrical chambers were used for photon beams, measurements of electron beams were performed using cylindrical as well as plane-parallel chambers. We observe that for the cylindrical chamber the maximum dose ratio discrepancy of the the DIN 6800-2 (1997) and TRS 398 (2000) with reference to DIN 6800-2 (DNS 2006) in photon beams varies between −0.4% to + 0.2% and in electron beams between −0.9% to + 1.1% respectively. With reference to DIN 6800-2 (DNS 2006) for the plane-parallel chamber, the discrepancy amounts to +0.5% for DIN 6800-2 (1997) and +1.5% for TRS 398 (2000).

Figure 1 shows that in general the values for photon beams are approximately the same for protocols DIN 6800-2 (DNS 2006) and TRS-398 (2000). A minor deviation can only be observed for the protocol DIN 6800-2 (1997) compared to the other two protocols.

The values in Figures 2 and 3 for electron beams show a higher difference than that for photon beams. While the values for the plane-parallel chamber according to the IAEA TRS 398 protocol show a tendency of relatively higher deviations, the values for the cylindrical chamber prove a better agreement for all protocols. In contrary to protocol IAEA TRS 398, the protocols of both DIN provide the formalism to calculate the quality correction factor k_E_. Therefore the cylindrical chambers can be used for the measurement of electron energies less than 10 MeV.

In an earlier work we compared protocols of IAEA TRS 398 and AAPM TG 51 against DIN 6800-2 (1997).[5] The discrepancies in the determination of absorbed dose to water for photon beams were within 1.0% and for electron beams 1.6%. The better agreement of this work in comparison to our earlier work can probably be explained by two facts. First, we have used Dosimeter system PTW-UNIDOS in the present work instead of the PTW-Dosimentor system. Secondly, the air density correction was done from the direct measurement of temperature and pressure instead of using the radioactive check source method. Moreover DIN 6800-2 (DNS 2006) protocol is used as a reference protocol.

The combined uncertainty for the determination of absorbed dose to water is calculated from the geometric summation of the different single uncertainties. Dosimetry protocol DIN 6800-2 (1997) does not mention any uncertainty estimation. But both DIN 6800-2 (DNS 2006) and IAEA TRS 398 protocols provide the method of evaluation of uncertainties for the determination of the quality correction factors. For all other correction factors we have estimated the respective uncertainties or they were taken from the instructions for use.

In Table 7 we present a list of factors that influence significantly the dosimetry and their respective uncertainties.

The maximal uncertainty for the determination of absorbed dose for photon and electron beams with the cylindrical chamber is estimated to be 1.25% following protocol TRS 398 and 1.42% for the DIN protocol. For the electron beam, the corresponding uncertainties are 0.96% and 1.50% for the *Roos* chamber. According to the German medical products law LMKM (2002)[6] the dosimetry protocol DIN 6800-2 is mandatory in Germany for the basic dosimetry.

The results following the protocol DIN 6800-2 (DNS 2006) have been checked with TLD inter-comparison by MTK. Our measurements following DIN 6800-2 (DNS 2006) showed deviations of less than 0.5% compared to the TLD measurements. An optimal level of quality assurance is assumed if the deviation is less than 3% (in Category A).

## Conclusion

DIN 6800-2 has been altered extensively and adopted to the IAEA TRS 398. In the protocols of IAEA and DIN 6800-2 (1997), cross-calibration is recommended for plane-parallel chambers against a calibrated cylindrical chamber, whereas in DIN 6800-2 (DNS 2006) experimental derived calibration factors are given for various types of plane-parallel chambers. Therefore the users do not need to perform the time-consuming and possibly inaccurate cross-calibration measurements. In the new DIN 6800-2 the evaluation of standard uncertainties of dose measurements is mentioned for the first time.

For electron beams, in DIN 6800-2 (DNS 2006) the measurement of the absorbed dose to water is recommended at the reference depth z_ref_, similar to protocol TRS 398. The characterization of the electron beam quality is a function of R_50_ instead of both quantities R_50_ and R_p_ as mentioned in the previous DIN protocol. In contrast to the IAEA protocol, the DIN protocol considers the quality correction factors for electron beams further on as a product of the two factors k_E_' and k_E_”.

The discrepancies in the determination of absorbed dose to water between the three protocols were 0.4% for photon beams and 1.5% for electron beams. For the measurement of electron beams with cylindrical chambers, the effective point of measurement is shifted according to all three protocols from the chamber axis towards the radiation source. The quality correction factor in the IAEA protocol is considered for the cylindrical chamber in electron beam including displacement correction factor k_r_, whereas that factor is not included in the quality correction factor of DIN. Therefore it should be considered separately.

For clinical dosimetry, our experiments showed that the time required is approximately the same for all three protocols. The advantage of the new protocol DIN 6800-2 (DNS 2006) lies in the renouncement of the cross-calibration as well as in its clear presentation of formulas and parameters.
